# Improving the Z_3_EV promoter system to create the strongest yeast promoter

**DOI:** 10.1093/femsyr/foae032

**Published:** 2024-10-18

**Authors:** Rina Higuchi, Yuri Fujita, Shotaro Namba, Hisao Moriya

**Affiliations:** Graduate School of Environmental, Life, Natural Science and Technology, Okayama University, Okayama 700-8530, Japan; Graduate School of Environmental, Life, Natural Science and Technology, Okayama University, Okayama 700-8530, Japan; Graduate School of Environmental, Life, Natural Science and Technology, Okayama University, Okayama 700-8530, Japan; Faculty of Environmental, Life, Natural Science and Technology, Okayama University, Okayama 700-8530, Japan

**Keywords:** yeast, overexpression, promoter

## Abstract

Promoters for artificial control of gene expression are central tools in genetic engineering. In the budding yeast *Saccharomyces cerevisiae*, a variety of constitutive and controllable promoters with different strengths have been constructed using endogenous gene promoters, synthetic transcription factors and their binding sequences, and artificial sequences. However, there have been no attempts to construct the highest strength promoter in yeast cells. In this study, by incrementally increasing the binding sequences of the synthetic transcription factor Z_3_EV, we were able to construct a promoter (P36) with ~1.4 times the strength of the *TDH3* promoter. This is stronger than any previously reported promoter. Although the P36 promoter exhibits some leakage in the absence of induction, the expression induction by estradiol is maintained. When combined with a multicopy plasmid, it can express up to ~50% of total protein as a heterologous protein. This promoter system can be used to gain knowledge about the cell physiology resulting from the ultimate overexpression of excess proteins and is expected to be a useful tool for heterologous protein expression in yeast.

## Teaser

The authors have developed the strongest *Saccharomyces cerevisiae* promoter to date, which can express a heterologous protein up to 50% of the total protein content.

## Introduction

Promoters play a central role in the artificial control of gene expression (Carey and Smale 2000). In the budding yeast *Saccharomyces cerevisiae*, various promoters have been constructed using endogenous gene promoters (Romanos et al. 1992, Weinhandl et al. 2014, Peng et al. 2015, Rajkumar et al. 2016), synthetic sequences (Vaishnav et al. 2022), synthetic transcription factors and their binding sequences (McIsaac et al. 2011, Azizoglu et al. 2021, Gligorovski et al. 2023). These promoters are characterized and utilized based on differences in expression strength, whether they are constitutive or controllable, and other factors. For controllable promoters, the method of control (such as temperature, drugs, light, etc.), the controllability (signal-to-noise ratio, minimum and maximum strength, etc.), and convenience (whether endogenous inducers are present or whether the promoter and control factor need to be introduced simultaneously) are important considerations. As for endogenous promoters, the *TDH3* promoter, known for its maximum expression strength, and the *GAL1* promoter, which can be repressed by glucose and induced by galactose, have been commonly used (Peng et al. 2015). Recently, there has been active development of synthetic promoters using artificial transcription activators/repressors and their binding sites. Examples include the WTC_846_ system (Azizoglu et al. 2021), which incorporates tet0 sites into the *TDH3* promoter and integrates feedback control of transcription factors, achieving tetracycline inducibility, high signal-to-noise ratio, wide dynamic range, and strong maximum expression strength; a promoter system that uses multiple binding sites for the synthetic transcription factor Z_3_EV (McIsaac et al. 2011, 2013, 2014), which can be induced by β-estradiol and avoids gratuitous transcription induction; and light-inducible promoter systems (Gligorovski et al. 2023). These promoters have been continuously improved to meet various criteria required for gene expression control. However, efforts to maximize expression strength have been limited.

In this study, we aimed to construct a promoter specifically designed to maximize the expression of recombinant proteins in yeast. Previously, we used the *TDH3* promoter to maximize the expression of excess proteins (Eguchi et al. 2018, Namba et al. 2022). However, the *TDH3* promoter had issues with its still insufficient strength, concerns about transcriptional competition with endogenous promoters especially when used on multicopy plasmids, and decreased expression in the post-diauxic phase due to its role as glycolytic proteins. To overcome these issues, we focused on the following conditions: (1) the promoter itself must have the highest transcriptional activation activity; (2) when the promoter is incorporated into a multicopy plasmid, the expression of other endogenous genes should not be affected by competition with transcription factors; (3) unintended proteins should not be expressed due to gratuitous induction; (4) the promoter should be minimally affected by the growth phase and do not need to change from the optimal growth conditions (i.e. glucose can be used as a primary carbon source). We thus considered that the Z_3_EV system promoter would match these conditions (McIsaac et al. 2013, 2014). The Z_3_EV system promoter is an artificial promoter constructed by modifying the *GAL1* promoter. This promoter is regulated by the synthetic transcription factor Z_3_EV and is induced by the drug β-estradiol. Z_3_EV is a composite protein consisting of a zinc-finger DNA-binding domain, the estrogen (β-estradiol) receptor, and the VP16 transcriptional activation domain. When β-estradiol binds to Z_3_EV, it moves into the nucleus and induces expression as a synthetic transcription factor (McIsaac et al. 2011). Because it is a completely synthetic system, it is expected that there will be a minimal reduction in the expression of other genes due to competition with transcription factors, and almost no gratuitous protein expression (McIsaac et al. 2013). Particularly, when integrating the comparative studies of the strongest promoters conducted so far (McIsaac et al. 2014, Kotopka and Smolke 2020, Gligorovski et al. 2023), the P3 promoter with six binding sites for the Z_3_EV promoter was considered the strongest. Attempts to increase the strength of this promoter using random mutagenesis with machine learning were made, but they were not very successful (Kotopka and Smolke 2020). On the other hand, attempts to increase the number of Z_3_EV binding sites have not been made. Therefore, in this study, we attempted to increase the strength by incrementally increasing the number of binding sites. As a result, the incremental increase in the number of Z_3_EV binding sites (up to 12) led to an increase in expression strength, achieving ~1.43 and 1.25 times the strength of the *TDH3* promoter and the P3 promoter, respectively. Increasing the number of binding sites beyond this point reduced the expression level. Although this promoter exhibited increased leakage in the absence of induction, transcriptional activation by β-estradiol was still maintained. Using this promoter with the combination of the gTOW multicopy plasmid system (Moriya et al. 2006, Moriya et al. 2012), we were able to express up to ~50% of the total protein as a heterologous protein. This promoter system can thus be used to gain knowledge about the cell physiology resulting from the ultimate expression of excess proteins and is a useful tool for heterologous protein expression in yeast.

## Materials and methods

The reagents, strains, plasmids, and primer sequences used in this study are summarized in the Table S1.

### Yeast growth conditions and transformation

The budding yeast strain DBY12394 (*MATα ura3Δ leu2Δ0::ACT1pr-Z3EV-NatMX*) was used (McIsaac et al. 2013). Yeast transformation was performed using the lithium acetate method (Amberg et al. 2005). Cells were cultured in synthetic complete (SC) medium (Amberg et al. 2005) without uracil (–Ura) or leucine and uracil (–LeuUra). All cultures were maintained at a temperature of 30°C.

### Plasmids used in this study

**Figure 1.**
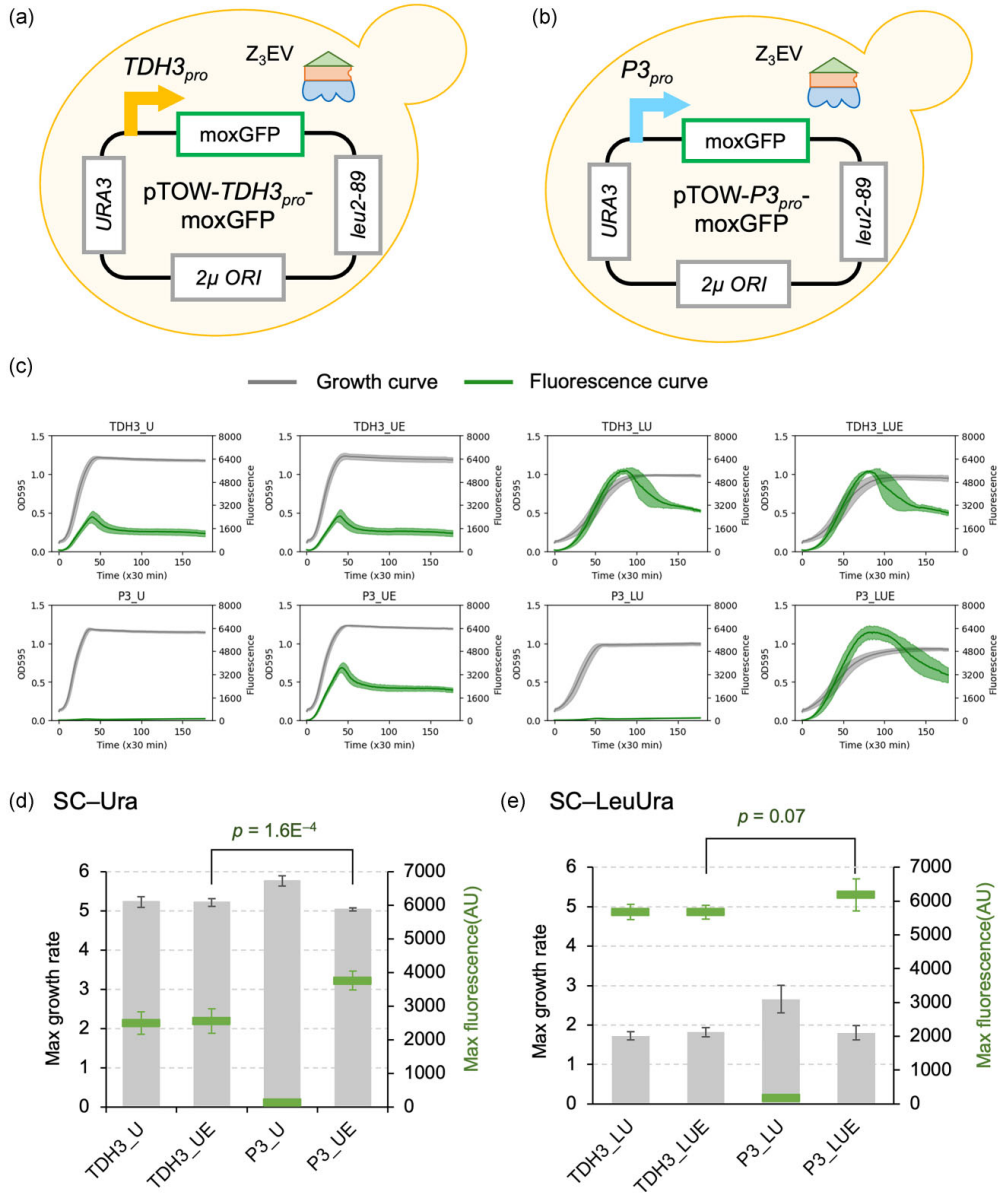
The P3 promoter exhibits strength comparable to the *TDH3* promoter. (a, b) Schematic diagrams of the cells used in this study. We used strains of DBY12394, which express the transcription factor Z_3_EV necessary for the P3 promoter, transformed with pTOW plasmids. MoxGFP expression is driven by the *TDH3* promoter (*TDH3_pro_*, a), or the P3 promoter (*P3_pro_*, b). (c) Growth curves and fluorescence values over time. The shaded areas represent the standard deviation. The left axis shows the turbidity of the culture measured at OD595, and the right axis shows the fluorescence intensity of moxGFP. (d, e) The maximum growth rate and max fluorescence intensity calculated from c. The bar graph represents the max growth rate, and the marker graph represents the max fluorescence intensity, with each error bar indicating standard deviation. Statistical tests were performed using Welch’s *t*-test (two-tailed). Measurements in c–e were performed using a fluorescence plate reader. In c, d, and e, “U,” “LU,” “UE,” and “LUE” represent SC–Ura, SC–LeuUra, SC–Ura+β-estradiol, and SC–LeuUra+β-estradiol, respectively. β-estradiol was added at a concentration of 1 µM. Experiments were performed with four or more biological replicas.

**Figure 2.**
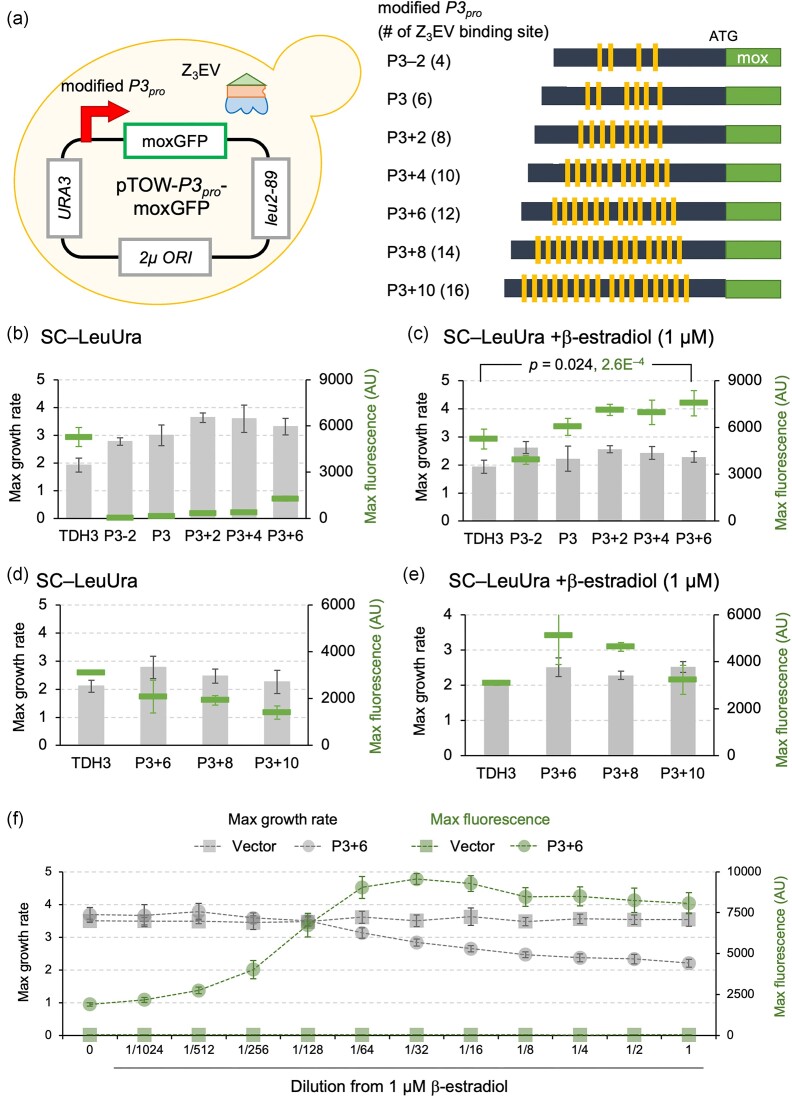
The effect of increasing Z3EV binding sites on transcriptional strength. (a) A schematic diagram of the cell used in the experiment and the structures of the modified P3 promoters. Blocks indicate the positions of the Z_3_EV binding sequences (gcgtgggcg). (b–e) Maximum growth rate (bar graph) and maximum fluorescence intensity (markers) in strains harboring plasmids with each promoter in SC–LeuUra medium (b and d) and SC–LeuUra medium with 1 µM β-estradiol added (c and e). In c, the *P*-values for the maximum growth rate and the maximum fluorescence intensity are indicated. (f) Induction of expression by β-estradiol for the P3 + 6 promoter. Maximum growth rate and maximum fluorescence intensity in SC–LeuUra medium with β-estradiol (diluted from 1 µM by half) are shown. (b–e) Experiments show the mean values and standard deviations (error bars) of four biological replicates measured by a fluorescence plate reader. Statistical tests were performed using Welch’s *t*-test (two-tailed).

**Figure 3.**
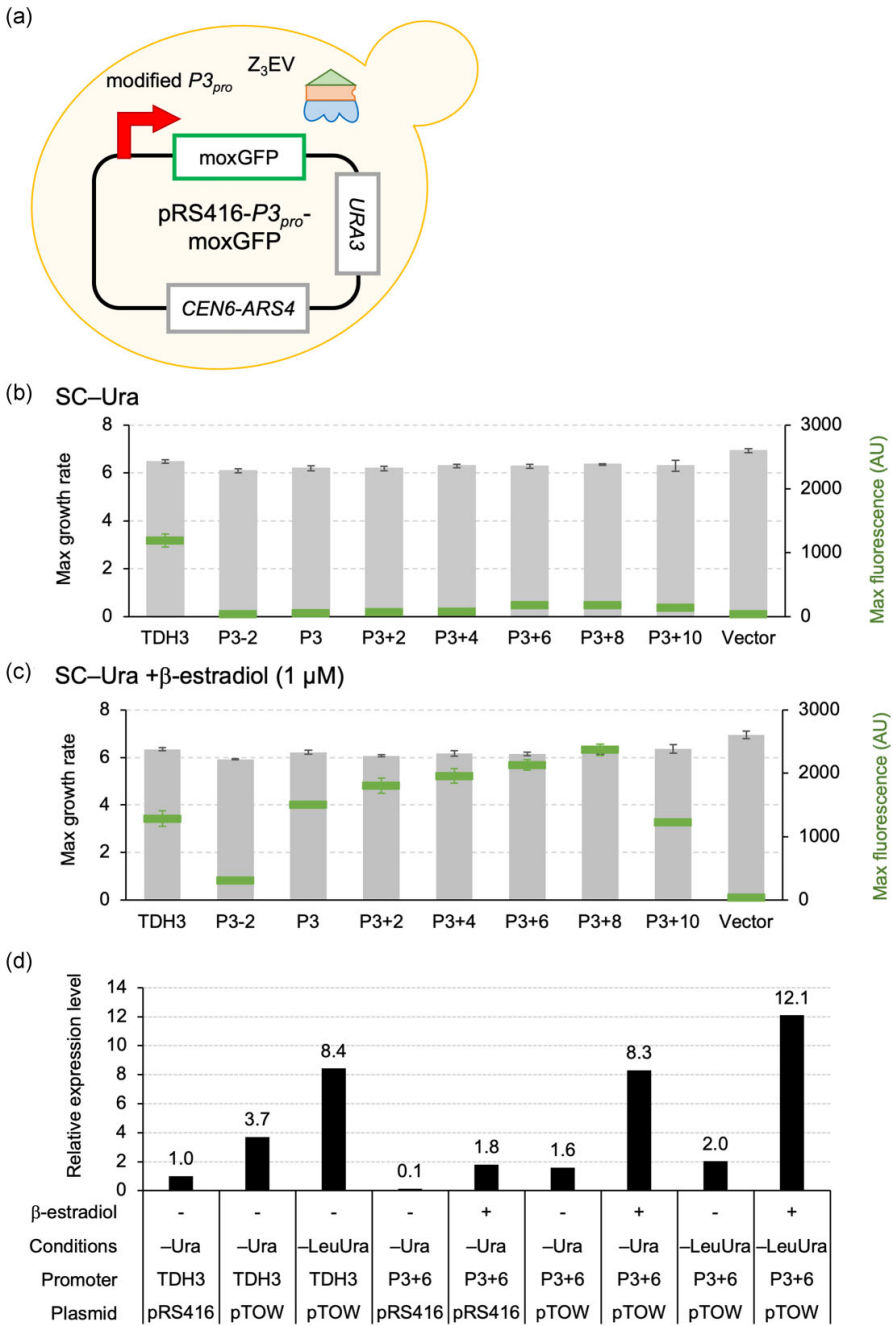
Evaluation of the strength of the modified P3 promoters on a low-copy plasmid. (a) A schematic diagram of the cell used in the experiment. (b–c) Maximum growth rate (bar graph) and maximum fluorescence intensity (markers) in strains harboring plasmids with each promoter in SC–Ura medium (b) and SC–Ura medium with 1 µM β-estradiol added (c). Experiments show the mean values and standard deviations (error bars) of four biological replicates measured by a fluorescence plate reader. (d) Comparison of the strength of the *TDH3* promoter and the P3 + 6 promoter across different plasmids, culture conditions, and with or without 1 µM β-estradiol induction. The maximum fluorescence intensity of moxGFP under the specified conditions was calculated as a relative expression level, with the strength of the *TDH3* promoter under the pRS416/SC–Ura condition set as 1.0.

**Figure 4.**
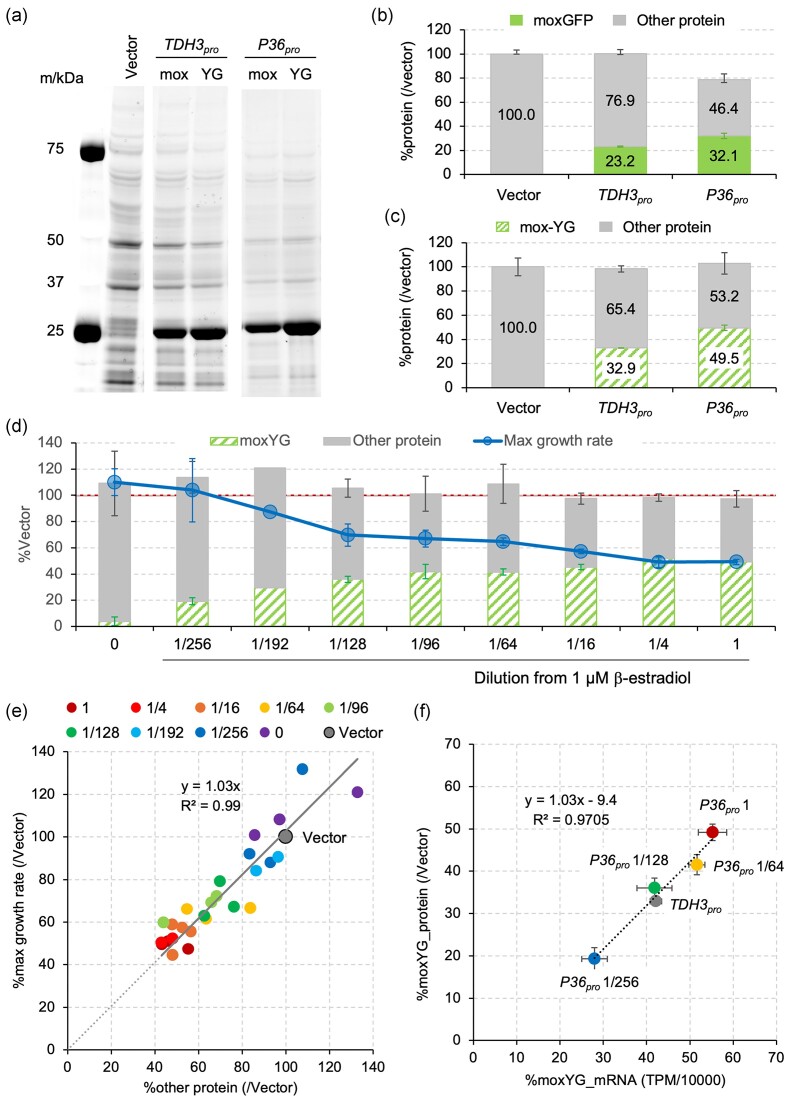
Maximum expression of excess protein using the P36 promoter. (a) Image of total proteins separated by SDS-PAGE. mox:moxGFP, YG:mox-YG. (b, c) Protein amounts measured from the SDS-PAGE gel images. The expression levels of moxGFP, mox-YG, and other proteins (Other protein) were calculated based on the total protein amount of the vector control, which was set to 100. The bar graphs show the average values obtained from three biological replicate experiments, with error bars indicating the standard deviation. (d) Maximum growth rate, mox-YG, and other protein amounts in cells expressing mox-YG from the P36 promoter with a stepwise dilution of β-estradiol. β-estradiol was added to SC–LeuUra medium in dilutions starting from 1 µM. The graph shows the averages of three biological replicates. The mean values and standard deviations (error bars) were calculated from three biological replicates, except for the 1/192 dilution, whose mean was calculated from two biological replicates. The dashed line indicates the total protein amount (100%) in vector control cells. (e) The relationship between maximum growth rate and protein amount in cells expressing mox-YG from the P36 promoter with a stepwise dilution of β-estradiol. All three biological replicates are shown as individual points, except for the 1/192 dilution (shown with two biological replicates). The regression line, its regression equation, and the *R*² value on a graph when performing linear regression through the origin are shown. (f) Comparison of mox-YG protein and mRNA levels. The mox-YG protein levels (same as in d) and mox-YG mRNA levels (calculated as percentages by dividing TPM values obtained from RNA-seq analysis by 10 000) are shown for the *TDH3* promoter (under SC–LeuUra conditions) and the P36 promoter (under SC–LeuUra conditions with varying β-estradiol concentrations from a 1 µM dilution). The mean values and standard deviations (error bars) were calculated from three biological replicates. The regression line, its regression equation, and the *R*² value on a graph when performing linear regression are shown. For a–e, the cultures and OD660 measurements were performed using a small shaking culture device.

Plasmids used in this study are listed in Table S1. In constructing the plasmid, synthetic DNA and polymerase chain reaction (PCR)-amplified DNA were joined using the recombination-based method in yeast (Oldenburg 1997), and their structures were verified by DNA sequencing. In the experiment shown in Fig. 3, the low-copy centromeric plasmid pRS416 (Sikorski and Hieter 1989) was used. The plasmids (pTOW) used in the experiments shown in Figs 1, 2, and 4 are high-copy plasmids with a *2µ ORI* and carry low-expression *LEU2* alleles (*leu2-89*). Therefore, after introducing these plasmids into *LEU2*-deficient strains and culturing in leucine-depleted media (SC–LeuUra), the plasmid copy number increases to over 100 copies. Due to the principle of the genetic tug-of-war (gTOW), the copy number of the plasmids increases to the level at which the target protein causes growth inhibition (Moriya et al. 2006, 2012).

### Measurement of growth and fluorescence

The promoter expression strength was evaluated using a reporter assay with moxGFP as the fluorescent protein reporter. Yeast cells were cultured statically under their respective medium conditions. For the measurements, a microplate reader (TECAN Infinite F200) was used to monitor and measure OD595 and Ex 485 nm/Em 535 nm every 30 min. The maximum growth rate was determined as described in the previous study (Moriya et al. 2006).

### Protein analysis and quantification

Yeast cells overexpressing the target protein were pre-cultured in SC–LeuUra medium, and then cultured in 5 ml of SC–LeuUra medium with or without β-estradiol using a shaking culture apparatus (ADVATEC, TVS062CA). Cells in the logarithmic growth phase (OD660 = 0.9–1.1) were treated with 1 ml of 0.2 N NaOH (Kushnirov 2000), followed by total protein extraction using 100 µl of LDS sample buffer (ThermoFisher). For each analysis, total protein was extracted from the amount of cells equivalent to 1.0 OD at OD660 (1 ODu). The extracted total proteins from 0.1 ODu cells were labeled with Ezlabel Fluoroneo (ATTO) according to the manufacturer’s protocol and separated by 4%–12% sodium dodecyl sulfate-polyacrylamide gel electrophoresis (SDS-PAGE). Protein detection and quantification were performed using the SYBR-green fluorescence detection mode of the LAS-4000 image analyzer (GE Healthcare) and Image Quant TL software (GE Healthcare). The total protein amount of the vector was set to 100%, and the amounts of moxGFP, mox-GY, and other proteins were quantified.

### RNAseq analysis

RNAseq analysis was performed essentially according to Namba et al. (Namba et al. 2022). The yeast was cultured in SC–LeuUra medium at 30°C and collected in the log phase (OD = 1.0–1.1). For the vector and *TDH3_pro_*-mox-YG, the culture was grown in medium without β-estradiol, while for *P36_pro_*-mox-YG, the culture was grown under induction conditions with β-estradiol diluted to 1/64, 1/128, and 1/256, based on the 1 µM concentration. RNA extraction was performed according to (Köhrer and Domdey 1991). The preparation of cDNA libraries and sequencing was outsourced to Macrogen, and conducted as follows: cDNA libraries were prepared using the TruSeq Stranded Total RNA kit (Illumina), and paired-end sequencing was performed using the Illumina Novaseq X system. Three biological duplications were analyzed for all strains. Sequences were checked for read quality by FastP (Chen et al. 2018) and aligned using Hisat2 (Kim et al. 2019). Aligned data were formatted into BAM files by Samtools (Li et al. 2009) and the amount of each transcript was quantified as TPM (Transcripts Per Million) using StringTie (Pertea et al. 2015). The raw data are available in the DNA Data Bank of Japan (accession number: PRJDB18827). The calculated TPM data are attached in Table S2.

## Results

### The P3 promoter exhibits strength comparable to the *TDH3* promoter

Because the P3 promoter has been reported to have strength equivalent to that of the *TDH3* promoter (Kotopka and Smolke 2020), we first verified whether the strength of the P3 promoter is indeed equivalent to that of the *TDH3* promoter using a fluorescent protein reporter assay. Specifically, we introduced the low-toxicity green fluorescent protein moxGFP (Namba et al. 2022) downstream of each promoter and constructed the pTOW plasmid (Fig. 1a, b). The pTOW plasmid utilizes the low-expression leucine synthesis enzyme gene (*leu2-89*) as a marker, which works as a selection bias to increase the plasmid copy number >100, allowing for maximum protein expression of the target protein on the plasmid when cultured in leucine-deficient SC–LeuUra medium (Moriya et al. 2006, 2012). Transcription from the P3 promoter is induced by 1 µM β-estradiol. Figure 1c shows the time-course changes in fluorescence and growth of each strain measured with a fluorescence plate reader. The maximum growth rate and maximum fluorescence level of each strain are shown in Fig. 1d and e. As expected, the *TDH3* promoter exhibited constitutive GFP expression with or without β-estradiol, while the P3 promoter showed significant GFP expression induction upon β-estradiol addition. Moreover, the maximum expression under the SC–LeuUra conditions was comparable to that of the *TDH3* promoter (Fig. 1e). Additionally, under SC–Ura conditions, the expression from the P3 promoter was significantly higher than that from the *TDH3* promoter (Fig. 1d). Therefore, it was confirmed that the P3 promoter has strength equal to or greater than that of the *TDH3* promoter.

### A promoter with 12 Z_3_EV binding sites (P36) shows the highest transcriptional activity

Z_3_EV-based promoters, including the P3 promoter, can change their strength by altering the position and number of Z3EV binding sites on the promoter (McIsaac et al. 2014). Therefore, we attempted to construct a promoter stronger than the *TDH3* promoter by increasing the number of Z_3_EV binding sites in the P3 promoter. Using the P3 promoter with 6 Z_3_EV binding sites as the base, we constructed new promoters with 4 binding sites, which is 2 fewer (P3-2), and incrementally added 2 binding sites at a time, up to a maximum of 16 binding sites (P3 + *n*, where n is 2 to 10) (Fig. 2a). The spacing of P3 was originally random, ranging from 22 bp to 28 bp (McIsaac et al. 2014), therefore for P3 + 2, we added two in the middle, and then we spaced the subsequent ones outward by 24 bp each. The constructed promoters were inserted into the pTOW plasmid and their strength was evaluated using a reporter assay, as described above. The results are shown in Fig. 2b–e.

As shown in Fig. 2d, under the maximum expression condition (SC–LeuUra+β-estradiol), the maximum fluorescence intensity increased with the increase in the number of Z_3_EV binding sites, and the P3 + 6 promoter was confirmed to have 1.43 and 1.25 times the strength of the *TDH3* promoter and the P3 promoter, respectively. Additionally, as shown in Fig. 2c, even under non-induced conditions (SC–LeuUra), the maximum fluorescence intensity increased with the increase in the number of Z_3_EV binding sites, indicating leakage expression with the increase in binding sites. On the other hand, when the number of Z_3_EV binding sites was increased to 14 (P3 + 8) and 16 (P3 + 10), the maximum fluorescence intensity decreased (Fig. 2e). From these results, it was suggested that the P3 + 6 promoter with 12 Z_3_EV binding sites is the strongest among the promoters constructed by modifying the P3 promoter. We note that the maximum expression level from the P3 + 6 promoter was higher than that of the *TDH3* promoter (*P* = 2.6E^−4^), while the maximum growth rate at this time was higher with the P3 + 6 promoter than with the *TDH3* promoter (*P* = 0.024, Fig. 2c). Therefore, it is suggested that the P3 + 6 promoter imposes a less extraneous burden, such as transcription factor competition, which the *TDH3* promoter potentially carries.

Next, we investigated the inducibility of the P3 + 6 promoter by β-estradiol (Fig. 2f). As predicted from Fig. 2c, there was leakage expression even in the absence of β-estradiol, but the expression level increased with the rise in β-estradiol concentration from zero to 1/32, and the inducibility was maintained with a maximum induction/non-induction ratio of 5.0. The addition of β-estradiol at concentrations of 1/64 (16 nM) or higher caused a decrease in cell growth. The fact that such growth reduction was not observed in the vector control, and that the degree of growth reduction became stronger with increasing β-estradiol concentration, suggests that the expression level increased stepwise from concentrations above 1/64 to 1 µM, causing growth inhibition due to the associated burden. Therefore, by using the P3 + 6 promoter, it is possible to investigate the effects on cells due to stepwise increases in expression levels, particularly in high-expression regions.

In the experiments conducted so far, we have aimed to induce gene expression as much as possible using the pTOW high-copy plasmid. However, because the copy number of high-copy plasmids can fluctuate within the cell, they are not suitable for strictly comparing promoter strengths. Therefore, we next evaluated promoter strength using the centromeric plasmid pRS416, which has a low and stable copy number (Sikorski and Hieter 1989). We transferred constructs containing the *TDH3* promoter as well as P3-2 to P3 + 10 promoters linked to moxGFP into pRS416 (Fig. 3a) and measured the growth rate and fluorescence intensity with and without β-estradiol induction (Fig. 3b and c). As a result, under induction conditions, P3 to P3 + 8 showed higher expression than *TDH3* (Fig. 3c). While P3 + 6 exhibited the highest strength in the high-copy plasmid (Fig. 2e), P3 + 8 showed the highest strength in the low-copy plasmid. Similar to the high-copy plasmid, P3 + 10 showed a decrease in expression strength. Therefore, while P3 + 6 was the strongest promoter in the multi-copy context (Fig. 2e), P3 + 8 is the strongest promoter when using a low-copy plasmid. In Fig. 3d, we summarized the relative expression levels of the *TDH3* promoter and the P36 promoter under various conditions, with the strength of the *TDH3* promoter on a low-copy plasmid set as 1.0. The combination of multi-copy amplification by pTOW and induction by the P3 + 6 promoter (hereafter referred to as the P36 promoter) resulted in up to 12-fold induction of expression compared to the single-copy *TDH3* promoter. This should be the strongest expression system in *S. cerevisiae* at present.

### Maximum expression of excess protein using the P36 promoter

In our recent study, we found that mox-YG, a mutated form of moxGFP that loses fluorescence, can be expressed in the highest amounts within yeast cells (Fujita et al. 2024). In that study, we used the combination of high-copy conditions of pTOW (SC–LeuUra) and the *TDH3* promoter. We then tested whether even more protein could be expressed using the P36 promoter. In Fig. 4a, the results of SDS-PAGE analysis of total proteins in cells during the logarithmic growth phase (OD660 = 0.9–1.1) in SC–LeuUra medium with the addition of 1 µM β-estradiol are shown. In Fig. 4b and c, the quantitative results of the expressed proteins are shown as the ratio of the target protein amount to the total protein amount in the vector control. The moxGFP expression level from the P36 promoter is 1.4 times that of the *TDH3* promoter, which closely matches the measurement of promoter strength by fluorescence (Fig. 4c). As expected, mutations that cause the loss of fluorescence increased protein expression levels in both promoters. Furthermore, expression from the P36 promoter was higher than from the *TDH3* promoter, achieving up to ~50% mox-YG expression. The total protein amount at this time was not different from the vector control (Fig. 4c). Therefore, the increase in mox-YG expression is directly reflected in the decrease in the amount of other proteins within the cell.

From previous experiments with *E. coli* and yeast, it is known that the increase in expression of excess proteins causes a gradual decrease in growth (Scott et al. 2010, Kafri et al. 2016). Therefore, we measured the growth rate and protein amount when the induction of mox-YG was gradually strengthened (Fig. 4d, e). As a result, strengthening the induction of mox-YG gradually decreased the growth rate. During this time, the total protein amount, including both mox-YG and other proteins, remained almost unchanged. Therefore, as the expression of mox-YG increased, the amount of other proteins decreased. Under these conditions, the decrease in maximum growth rate and the decrease in the amount of proteins other than mox-YG (Other protein) could be approximated as a straight line through the origin with a slope close to 1 (1.03), and the *R*² value was 0.99 (Fig. 4e). From these results, it was found that the P36 promoter has the maximum strength to reach 50% of the total protein and allows for gradual regulation of protein expression.

Finally, we quantified the mox-YG mRNA level from the P36 promoter at each β-estradiol concentration. The results showed that the mRNA levels increased in a concentration-dependent manner and exhibited a linear relationship with the protein levels (Fig. 4f). Even at the maximum level of expression induction (1 µM β-estradiol), the linear relationship between mRNA and protein levels remained intact, suggesting that the cell’s translational capacity is not saturated under these conditions. Therefore, it is implied that further increasing the mRNA levels could potentially lead to an even greater increase in protein production.

## Discussion

In this study, we aimed to construct an artificial promoter with high expression strength by increasing the number of Z_3_EV binding sites. The P36 promoter, which has six additional binding sites in the P3 promoter, was the strongest among those constructed, with a strength 1.4 times that of the *TDH3* promoter and 1.2 times that of the P3 promoter under high-copy plasmid conditions (Fig. 2c). Although there was significant leakage without β-estradiol addition, the inducibility was maintained at ~5-fold (Fig. 2f). Under low-copy conditions, P3 + 8 exhibited the highest expression (Fig. 3c). In high-copy conditions, the difference in strength between P36 and P3 + 8 might not be apparent (Fig. 2e) due to the depletion of transcription factors. When the number of binding sites was further increased, decreases in strength were observed (Figs 2d, e, 3b, and c). The reasons for these decreases could be that the Z3EV binding sites are too close to each other or to the transcription start sites, causing interference. Different configurations of the binding sites might further increase promoter strength.

The P36 promoter is considered to be a very powerful tool for studying the growth inhibition effects (protein burden) caused by the overproduction of excess proteins. In *E. coli* studies, there is a linear relationship between the expression of excess proteins and growth reduction, with an estimate that expressing around 36% excess protein results in growth cessation (Bruggeman et al. 2020). A similar linear relationship has been observed in yeast cells, but the amount of excess protein that can be expressed was not as high (Kafri et al. 2016). One of the reasons for this is considered to be the insufficient strength of the promoters. Using the P36 promoter developed in this study with the high-copy gTOW system, it is possible to express up to 50% excess protein (Fig. 4c), and through stepwise induction, a wide range of linear relationships similar to those seen in *E. coli* were observed (Fig. 4e). Interestingly, yeast cells expressing 50% excess protein maintained a 50% growth rate, with the origin of the regression line being zero (Fig. 4e). This suggests that eukaryotic cells have a higher capacity to accommodate excess proteins compared to prokaryotic cells and that prokaryotic and eukaryotic cells may have different response regimes to protein burden.

Finally, in this study, we constructed promoter plasmids capable of inducing expression across a wide range of levels, both in low-copy and high-copy contexts. These promoter plasmid resources can be utilized to explore and achieve optimal expression levels, particularly in the high-expression range, for both homologous and heterologous protein expression.

## Supplementary Material

foae032_Supplemental_Files

## Data Availability

The RNA-seq raw data are available in the DNA Data Bank of Japan (accession number: PRJDB18827). The calculated TPM data are provided in Table S2. The raw data used to create the graphs are provided as Data S1.

## References

[bib1] Amberg DC, Burke DJ, Burke D et al. Methods in Yeast Genetics: a Cold Spring Harbor Laboratory Course Manual. New York: CSHL Press, 2005.

[bib2] Azizoglu A, Brent R, Rudolf F. A precisely adjustable, variation-suppressed eukaryotic transcriptional controller to enable genetic discovery. eLife. 2021;10:e69549. 10.7554/eLife.69549. 34342575 PMC8421071

[bib3] Bruggeman FJ, Planqué R, Molenaar D et al. Searching for principles of microbial physiology. FEMS Microbiol Rev. 2020;44:821–44. 33099619 10.1093/femsre/fuaa034 PMC7685786

[bib4] Carey MF, Smale ST. Transcriptional Regulation in Eukaryotes: Concepts, Strategies, and Techniques. New York: CSHL Press, 2000.

[bib5] Chen S, Zhou Y, Chen Y et al. fastp: an ultra-fast all-in-one FASTQ preprocessor. Bioinformatics. 2018;34:i884–90. 30423086 10.1093/bioinformatics/bty560 PMC6129281

[bib6] Eguchi Y, Makanae K, Hasunuma T et al. Estimating the protein burden limit of yeast cells by measuring the expression limits of glycolytic proteins. eLife. 2018;7:e34595. 10.7554/eLife.34595. 30095406 PMC6086662

[bib7] Fujita Y, Namba S, Moriya H. Impact of maximal overexpression of a non-toxic protein on yeast cell physiology. 2024. 10.7554/elife.99572 PMC12443478 40960085

[bib8] Gligorovski V, Sadeghi A, Rahi SJ. Multidimensional characterization of inducible promoters and a highly light-sensitive LOV-transcription factor. Nat Commun. 2023;14:3810. 37369667 10.1038/s41467-023-38959-8 PMC10300134

[bib9] Kafri M, Metzl-Raz E, Jona G et al. The cost of protein production. Cell Rep. 2016;14:22–31. 26725116 10.1016/j.celrep.2015.12.015 PMC4709330

[bib10] Kim D, Paggi JM, Park C et al. Graph-based genome alignment and genotyping with HISAT2 and HISAT-genotype. Nat Biotechnol. 2019;37:907–15. 31375807 10.1038/s41587-019-0201-4 PMC7605509

[bib11] Köhrer K, Domdey H. [27]Preparation of high molecular weight RNA. Methods Enzymol. 1991;194:398–405. 1706459 10.1016/0076-6879(91)94030-g

[bib12] Kotopka BJ, Smolke CD. Model-driven generation of artificial yeast promoters. Nat Commun. 2020;11:2113. 32355169 10.1038/s41467-020-15977-4 PMC7192914

[bib13] Kushnirov VV. Rapid and reliable protein extraction from yeast. Yeast. 2000;16:857–60. 10861908 10.1002/1097-0061(20000630)16:9<857::AID-YEA561>3.0.CO;2-B

[bib14] Li H, Handsaker B, Wysoker A et al. The sequence alignment/map format and SAMtools. Bioinformatics. 2009;25:2078–9. 19505943 10.1093/bioinformatics/btp352 PMC2723002

[bib15] McIsaac RS, Gibney PA, Chandran SS et al. Synthetic biology tools for programming gene expression without nutritional perturbations in Saccharomyces cerevisiae. Nucleic Acids Res. 2014;42:e48. 24445804 10.1093/nar/gkt1402 PMC3973312

[bib16] McIsaac RS, Oakes BL, Wang X et al. Synthetic gene expression perturbation systems with rapid, tunable, single-gene specificity in yeast. Nucleic Acids Res. 2013;41:e57. 23275543 10.1093/nar/gks1313 PMC3575806

[bib17] McIsaac RS, Silverman SJ, McClean MN et al. Fast-acting and nearly gratuitous induction of gene expression and protein depletion in Saccharomyces cerevisiae. MBoC. 2011;22:4447–59. 21965290 10.1091/mbc.E11-05-0466 PMC3216669

[bib18] Moriya H, Makanae K, Watanabe K et al. Robustness analysis of cellular systems using the genetic tug-of-war method. Mol BioSyst. 2012;8:2513–22. 22722869 10.1039/c2mb25100k

[bib19] Moriya H, Shimizu-Yoshida Y, Kitano H. In vivo robustness analysis of cell division cycle genes in Saccharomyces cerevisiae. PLoS Genet. 2006;2:e111. 16839182 10.1371/journal.pgen.0020111 PMC1500812

[bib20] Namba S, Kato H, Shigenobu S et al. Massive expression of cysteine-containing proteins causes abnormal elongation of yeast cells by perturbing the proteasome. G3. 2022;12:jkac106. 10.1093/g3journal/jkac106. 35485947 PMC9157148

[bib21] Oldenburg K. Recombination-mediated PCR-directed plasmid construction in vivo in yeast. Nucleic Acids Res. 1997;25:451–2. 9016579 10.1093/nar/25.2.451 PMC146432

[bib22] Peng B, Williams TC, Henry M et al. Controlling heterologous gene expression in yeast cell factories on different carbon substrates and across the diauxic shift: a comparison of yeast promoter activities. Microb Cell Fact. 2015;14:91. 26112740 10.1186/s12934-015-0278-5 PMC4480987

[bib23] Pertea M, Pertea GM, Antonescu CM et al. StringTie enables improved reconstruction of a transcriptome from RNA-seq reads. Nat Biotechnol. 2015;33:290–5. 25690850 10.1038/nbt.3122 PMC4643835

[bib24] Rajkumar AS, Liu G, Bergenholm D et al. Engineering of synthetic, stress-responsive yeast promoters. Nucleic Acids Res. 2016;44:e136. 27325743 10.1093/nar/gkw553 PMC5041464

[bib25] Romanos MA, Scorer CA, Clare JJ. Foreign gene expression in yeast: a review. Yeast. 1992;8:423–88. 1502852 10.1002/yea.320080602

[bib26] Scott M, Gunderson CW, Mateescu EM et al. Interdependence of cell growth and gene expression: origins and consequences. Science. 2010;330:1099–102. 21097934 10.1126/science.1192588

[bib27] Sikorski RS, Hieter P. A system of shuttle vectors and yeast host strains designed for efficient manipulation of DNA in saccharomyces cerevisiae. Genetics. 1989;122:19–27. 2659436 10.1093/genetics/122.1.19 PMC1203683

[bib28] Vaishnav ED, de Boer CG, Molinet J et al. The evolution, evolvability and engineering of gene regulatory DNA. Nature. 2022;603:455–63. 35264797 10.1038/s41586-022-04506-6 PMC8934302

[bib29] Weinhandl K, Winkler M, Glieder A et al. Carbon source dependent promoters in yeasts. Microb Cell Fact. 2014;13:5. 24401081 10.1186/1475-2859-13-5 PMC3897899

